# Books and Authors

**Published:** 1910-03

**Authors:** 


### BOOKS AND AUTHORS.

---

**Primer of Sanitation.** Disease Germs and How to Fight Them. By **John W. Ritchie,** Professor of Biology, College of William and Mary, Virginia. 12mo, pp. 206. Illustrated by **Karl Hassmann.** Yonkers: World Book Co. (Cloth, 50 cents.)

Professor Ritchie has already been introduced to the readers of the Journal through his school physiology, a notice of which was published in the October, 1909, number. This primer of sanitation is the first book in English to offer fifth or sixth grade pupils the advantages of knowledge of germ diseases and how to escape them. The author discourses upon his subject in simple yet forceful language, and at the end of each chapter under several heads, groups the points to be remembered. More than one hundred illustrations serve to impress the facts upon the young mind, though the author is in error in saying Dr. Walter Reed allowed himself to be bitten by infected mosquitoes, thus contracting yellow fever from which he died.

**The Practical Medicine Series.** Ten volumes. Under the general editorial charge of **Gustavus P. Head,** M.D., Professor of Laryngology and Rhinology in the Chicago Post-Graduate Medical School. Vol. IX. Skin and Venereal Diseases and Miscellaneous Topics. Edited by **W. L. Baum,** M.D., and **Harold N. Moyer,** M.D. Series 1909. Chicago. The Year Book Publishers. (Price, $1.25; entire series, $10.00.)

The section on skin and venereal diseases in this volume is prepared by William L. Baum. Pellagra receives first attention, a succinct account of the disease as observed in this country being given. It is interesting to learn that the *x*-rays destroy the sweat glands, therefore their use in axillary sweating effect a cure. The venereal diseases are effectively considered, many newer methods of cure being recorded.

In the second section Harold N. Moyer gives a group of miscellaneous abstracts of interest. They embrace heredity, longevity, necessity of autopsies, body weight, medico-ethical problems, criminal abortion, birth rate, and many other topics of interest and importance.

---

**A Textbook of Physiology:** for medical students and physicians. By **William H. Howell,** Ph.D., M.D., LL.D., Professor of Physiology, Johns Hopkins University, Baltimore. Third edition. Revised. Octavo of 998 pages, illustrated. Philadelphia and London: W. B. Saunders Company. 1909. (Cloth, $4.00; half morocco, $5.50 net prices.)

The first edition of this work was printed in September, 1905, the second in August, 1907, and this, the third has just been put forth. Since the second edition was sent out considerable material has been added to the literature of the subject, furnishing the author a large field from which to glean facts of much value. Physical physiology in particular has developed to quite an extent of late, and the facts relating thereto have engaged the author's attention. Those facts of importance have been incorporated in this work. That the author is an enthusiast is discovered before one has gone far in examining his treatise; that he has prepared it with care is another fact that sinks deep into the mind as the work is examined more closely; and that it is one of the best of the textbooks on physiology extant, is an impression that it leaves after the examination is completed.

---

**A Textbook upon the Pathogenic Bacteria.** By **Joseph McFarland,** M.D., Professor of Pathology and Bacteriology in the Medico-Chirurgical College, Philadelphia. Sixth edition. Octavo of 709 pages, illustrated. Philadelphia and London. W. B. Saunders Company. 1909. (Cloth, $3.50 net.)

The author has explained that this is a textbook on pathogenic bacteria, but does not include the non-pathogenic, nor the pathogenic protozoa. It is one of the most complete works of its kind yet in print, and is a guide for student, teacher, and practitioner, embracing the very latest knowledge on the subject with which it deals. The bacteriology of food is an interesting study, not to say an important one at the present time, in view of the action of the government in an attempt to protect the people against impure food and drugs. In revising the material for this, the sixth edition, the author has assembled a vast number of references, a task of great labor, but of incalculable benefit to the student who would examine original writings that have become classics, and which contain the latest experimental work on the topics dealt with.

---

**A Textbook on the Practice of Gynecology.** By **W. Easterly Ashton**, M.D., LL.D., Professor of Gynecology in the Medico-Chirurgical College of Philadelphia. Fourth edition. Octavo of 1099 pages, with 1058 original line drawings. Philadelphia and London: W. B. Saunders Company. 1909. (Cloth, $6.50; half morocco, $8.00, net prices.)

This author and his treatise have become well acquainted with the profession,—the book through the three previous editions, the author through his teachings at the Medico-Chi. and through his book which was first issued in 1905. In the preface to the first edition Ashton tells that he aims to take nothing for granted in describing gynecologic diseases, by which we suppose he means to tell only of what he has seen and of that which he absolutely knows. We do not intend to pursue the author's methods in detail at this time, that having been done heretofore; but we invite attention to this statement that the reader who may not have seen the previous editions may understand the matter.

Ashton is a gynecologist of experience and a teacher who gives force and impact to his words as well as clearness to his expressions. His sentences are not involved and his teachings are readily comprehended. He has made the present edition embrace the advances made in gynecology that have proved worthy, since the last previous edition was issued. New material has been inserted here and there; as heretofore, he lays great stress on the importance of indoor exercise, and he accentuates the effects of wearing corsets. The illustrations, all drawn from nature, are original and effective. The treatise is practical and an excellent setting forth of the present status of gynecology.

---

**Surgery:** Its Principles and Practice. In five volumes. By 66 eminent surgeons. Edited by **W. W. Keen**, M.D., LL.D., Hon. F.R.C.S., Eng. and Edin., Emeritus Professor of the Principles of Surgery and of Clinical Surgery, Jefferson Medical College, Phila. Volume V. Octavo of 1274 pages, with 550 illustrations, 45 in colors. Philadelphia and London. W. B. Saunders Company. 1909. (Per volume; cloth, $7.00; half morocco, $8.00, net prices.)

This volume, the fifth and last, of Keen's surgery deals with surgery of the vascular system by Rudolph Matas; surgery of the female genitourinary organs, by E. E. Montgomery, John M. Fisher and P. Brooke Bland; surgical technic, by John H. Gibbon; ligation of arteries in continuity, by Warren Stone Bickham; operations on bones and joints, by James Peter Warbasse; amputations, by Warren Stone Bickham; plastic or reconstructive surgery, by John B. Roberts; surgery of accidents, by William L. Estes; surgery of the parathyroid bodies, by Charles H. Mayo; intracranial surgery of the fifth (trigeminal) and the eighth (auditory) nerves, by Charles H. Frazier; general anesthesia and anesthetics, by Hobart Amory Hare; local and subarachnoid (spinal) anesthesia, by Karl G. Lennander and Fredrik Zachrisson; surgery of the infectious diseases, by George E. Armstrong; use of the *x*-ray and radium in surgery, by Ernest Amory Codman; legal relations of the surgeon, by Hampton L. Carson, Esq.; the laboratory as an aid to surgical technic and surgical diagnosis, by William M. Late Coplin; the surgical organisation of a hospital, by Albert J. Ochsner. Each one of the foregoing titles relates to surgical topics of the highest importance. To those interested in head surgery we direct attention to the chapters pertaining to that important branch of surgical procedures and in particular, to an addendum prepared by Dr. Keen, himself, relating to the means of opening the skull, in which the instruments devised by Hudson, of Atlanta, are depicted. Keen says they combine the safety of the chisel and the speed of the various mechanical saws whether driven by hand or power. Besides the burrs, Hudson has devised an osteoplastic forceps which is useful in making an osteoplastic flap. These instruments greatly simplify much of the technic of head surgery.

It is manifestly unnecessary to make extended comment on this important work which has already been in the eye of the profession for more than two years. It is competent, however, for us in closing this notice to remark that it is doubtful whether any surgical treatise has been delivered into the hands of the profession which has set forth the surgical art of the present day in a more comprehensive or scientific manner than Keen's surgery.

